# Mechanical, Electrical, and Piezoresistive Sensing Characteristics of Epoxy-Based Composites Incorporating Hybridized Networks of Carbon Nanotubes, Graphene, Carbon Nanofibers, or Graphite Nanoplatelets

**DOI:** 10.3390/s20072094

**Published:** 2020-04-08

**Authors:** XiaoDong Wang, JianChao Wang, Swarup Biswas, Hyeok Kim, IlWoo Nam

**Affiliations:** 1 College of Civil Engineering, Nanjing Tech University, 30 Puzhu Road(S), Nanjing 211800, China; xd.wang@njtech.edu.cn (X.W.); jc.wang@njtech.edu.cn (J.W.); 2 School of Electrical and Computer Engineering, University of Seoul, 163 Seoulsiripdaero, Dongdaemun-gu, Seoul 02504, Korea; biswas.swarup1988@gnu.ac.kr (S.B.); hyeok.kim@uos.ac.kr (H.K.); 3 School of Spatial Environment System Engineering, Handong Global University, 558 Handong-ro, Buk-gu, Pohang, Gyeongbuk 37554, Korea

**Keywords:** carbon nanotubes and nanofibers, graphene, piezoresistive sensing, pressure sensing

## Abstract

The present study compared the mechanical, electrical, morphological, and piezoresistive characteristics of epoxy-based sensing nanocomposites fabricated with inclusions of hybridized networks of four different carbon nanomaterials (CNMs), such as carbon nanotube (CNT), graphene, carbon nanofiber (CNF), and graphite nanoplatelet (GNP). Enhancements in elastic modulus and electrical conductivity were achieved by CNT–graphene composites and CNT–CNF composites, and these were explained by the morphological observations carried out in the present study and experimental studies found in the literature. The greatest gauge factor was accomplished by the CNT–GNP composite, followed by the CNT–CNF composite among composites where the CNM networks were sufficiently formed with a content ratio of 3%. The two types of the composites outperformed the composites incorporating solely CNT in terms of gauge factor, and this superiority was explained with the excluded volume theory.

## 1. Introduction

Polymer composites with multifunctionality recently became a research topic of interest for numerous global researchers in various fields such as mechanical characteristics reinforcing, stress/strain sensing, electromagnetic wave shielding/absorbing, and others [1,2,3,4]. The research fields mentioned require the mechanical and electrical characteristics of composites to meet a certain standard, and it is imperative that conductive materials such as carbon or metal materials are incorporated into an insulating polymer matrix in order to produce composites with the necessary electrical characteristics [5].

As an inclusion material in polymer composites, carbon nanomaterials (CNMs) have been actively utilized by many researchers in the last two decades. CNMs are classified into several types according to shape (geometry), dimension, and nanostructural features, and the principal CNMs are carbon nanotube (CNT), graphene, carbon nanofiber (CNF), and graphite nanoplatelet (GNP) [6]. While CNT and CNF have a common geometrical feature in that both types of materials have a one-dimensional shape of a fiber, there are significant differences in the dimensions of the two materials. The diameter of CNT rises to tens of nanometers, but the diameter of CNF is as large as hundreds of nanometers [3]. With graphene and GNP, the common features include both types being composed of graphitic layers and having a planar two-dimensional shape, but there are differences in the number of the layers [3,7]. The number of layers for graphene is known to be one or few, but GNP is composed of tens of layers [8,9].

CNMs, CNTs, graphene, CNFs, and GNPs all possess excellent mechanical characteristics, and it has been reported that the CNMs brought enhancements to the mechanical characteristics of polymer composites [1,3]. In addition, the CNMs have electrically conductive characteristics due to π-electron systems (*sp*^2^ orbitals) in carbon atoms [10,11]. In particular, CNT and graphene are known to have outstanding electrical conductivity comparable to or greater than that of copper wire. It has been said in studies in the literature that enhancements in electrical characteristics were accomplished by embedding CNMs in polymer matrices [5,12].

The incorporation of CNMs in polymer matrices not only produced enhancements in the mechanical/electrical characteristics but also enabled the resultant polymer composites to sense stress/strain resulting from external loadings [13]. This stress/strain sensing of the composites is manifested by means of the coupling of electrical properties and mechanical deformation of the CNMs network, and the phenomenon of changes in mechanical characteristics leading to changes in electrical characteristics refers to the piezoresistive characteristics [1]. The piezoresistive characteristics of a CNM network are closely related to three factors: (1) the disruption and formation of the conductive connections in CNMs, (2) changes in the distance between CNMs, and (3) deformation of the CNM itself [14]. For example, if a tensile loading is applied to a CNM-incorporated composite, the interconnections of the CNMs are disrupted in local parts of the CNM network, and the distance between CNMs becomes larger. This, in turn, leads to the deterioration of the electrical properties of the composite [15].

Due to their mechanical, electrical and piezoresistive characteristics, CNMs are being utilized in the development of polymer composites that require excellent mechanical and electrical characteristics, and they can be used as key materials in the development of composites with multifunctionality, such as mechanical characteristics reinforcing and piezoresistive sensing [1,3,16,17,18].

However, several researchers have reported drawbacks, such as limited mechanical and electrical properties and low sensing performance when the composites were fabricated using a single type of CNM [19,20]. To eliminate these drawbacks, a hybridized CNMs network was introduced, and studies of its mechanical, electrical and piezoresistive characteristics were started a few years ago [1,21,22,23]. Several studies have reported synergistic effects, indicating further enhancements that could not be accomplished using a single type of CNM under the same conditions [1,24,25]. This was demonstrated in terms of mechanical, electrical, and piezoresistive characteristics by means of hybridized CNT–graphene networks [1,24,25]. However, attempts to harness the outperforming hybridized networks in the development of multifunctional polymeric composites have been scarce.

In this regard, the present study examined the mechanical, electrical, morphological and piezoresistive characteristics of epoxy composites that incorporated a hybridized CNM network. Among the four types of CNMs, which are CNT, graphene, CNF and GNP, two types were selected to make different sets of hybridized networks, and the composites fabricated with the hybridized networks were compared with CNT-only embedded composites. The mechanical and electrical characteristics of the fabricated composites were assessed in terms of tensile elastic modulus and direct current (DC) conductivity, respectively. In addition, the piezoresistive characteristics were assessed in terms of gauge factor and stability factor. Characterization and comparison studies of CNM-embedded epoxy composites provide advancing knowledge for the development of multifunctional polymer composites possessing mechanical, electrical, and piezoresistive characteristics that cannot be obtained with composites incorporating only a single type of CNM.

## 2. Materials and Methods

Multi-walled carbon nanotube (MWNT), CNF, and graphene purchased from Beijing Daoking Co. Ltd. (Beijing, China) and GNP purchased from Timenano Co. Ltd. (Chengdu, China) were used in the present study. The dimensions and material properties of the CNMs are listed in Table 1. An epoxy was composed of a 3:1 mix ratio of epoxy resin (E-4676) and hardener (HC-3008-5), from Kunshan Xiangfeng New Composite Co., Ltd. (Kunshan, China).

The diameter and length of CNTs, graphene, and GNP were estimated by observation of SEM images provided from manufacturers and the SEM images can be found in Figures S1, S2, and S3. The specific surface area of CNTs, graphene, and CNF was determined by the Brunauer-Emmett-Teller (BET) method. The electrical conductivity of the CNTs and graphene was determined by measuring the electrical resistance of the two materials, respectively, in the dried powder status. The resistance of the dried CNM powder was derived by employing Ohm’s law and calculated by dividing the voltage potential produced in the CNM by a constant electric current of 10 mA supplied to the CNM under temperature of 23 °C and relative humidity of 40–65%.

To study the mechanical, electrical and piezoresistive characteristics of epoxy composites incorporating CNMs, various mix proportions were adopted for composite fabrication. A group of composites named “CNT-only composites” were fabricated using only CNTs, and the weight proportion of CNT was varied as 1%, 1.5%, or 3% by total weight of CNT/epoxy mixture. The CNT-only composites were employed as a reference group because these composites showed the best electrical properties in a preliminary test that was carried out to examine the electrical characteristics of composites incorporating a single type of CNM. In addition to the CNT-only composites, three groups of composites incorporating a hybridized CNMs network—CNT–graphene, CNT–CNF and CNT–GNP—were also fabricated and used in weight proportions of 1%, 1.5%, and 3% by total weight of the mixture. A filler proportion of the two CNMs used in an identical hybridized network was 1:1, and this was consistent in the three groups of composites [26]. Moreover, composites incorporating only graphene with content ratios of 1% and 1.5% were fabricated for use in a comparison study of electrical conductivity, and in a similar fashion, CNF-only composites and GNP-only composites were also fabricated with content ratios of 1% and 1.5%.

**Figure 1:**
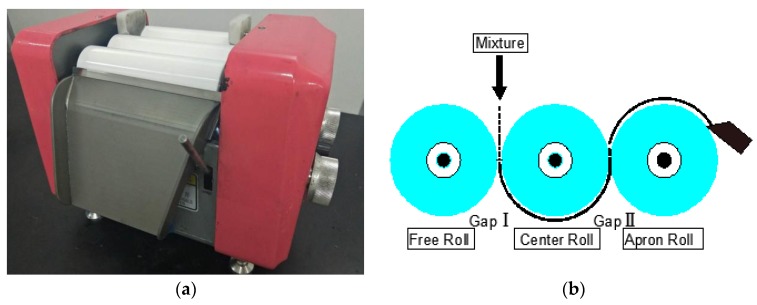
(**a**) Three-roll milling machine for dispersing CNM in the composites, and (**b**) a schematic diagram illustrating dispersion procedures implemented by three-roll milling machine.

The sample preparation method was as follows. Each component of the epoxy resin, hardener, and CNM were weighed according to the mix proportion and poured into a bowl. The mixture was gently stirred by hand for 2 min. After the premixing, a three-roll milling machine (ZYTR-50, Shenzhen Zhong Yi Technology Co. Ltd., Shenzhen, China) was employed to obtain even distribution of the CNMs in the composites. The schematic diagram of the three-roll milling process shown in Figure 1 illustrates three rolls rotating in parallel and two gaps (Gap I and Gap II) produced by the three rolls. The mixture was poured into Gap I, and it was then drawn off at Gap II as the three rolls rotated. In the course of the milling, the shear force exerted by the rotating roller facilitated the distribution of CNMs in the epoxy resin. The pouring and withdrawal cycle of the mixture was carried out 12 times per batch, and the sizes of Gaps I and II were adjusted in each milling cycle according to Table 2. Subsequently, the final mixture was decanted to a polytetrafluoroethylene (PTFE) mold with dimensions that complied with the standards of type IV in ASTM D638. After one day of curing, the samples were demolded.

The electrical conductivity was determined from a parallelepiped sample with dimensions of 3 mm × 20 mm × 20 mm that was measured by means of a digital multimeter (Keysight DMM 34461A; Keysight Technologies, Santa Rosa, CA, USA). Silver paste was applied to the sides of the sample that faced in opposite directions and used as an electrode. Electrical conductivity was determined using the following formula [27,28],

(1) $$\sigma =\frac{L}{RA}$$

where σ (S/m), *R* (Ohm), *L* (mm), and *A* (mm^2^) denote the DC conductivity, DC resistance, distance between the two electrodes of the sample, and cross-sectional area of the sample, respectively [27,28].

**Figure 2:**
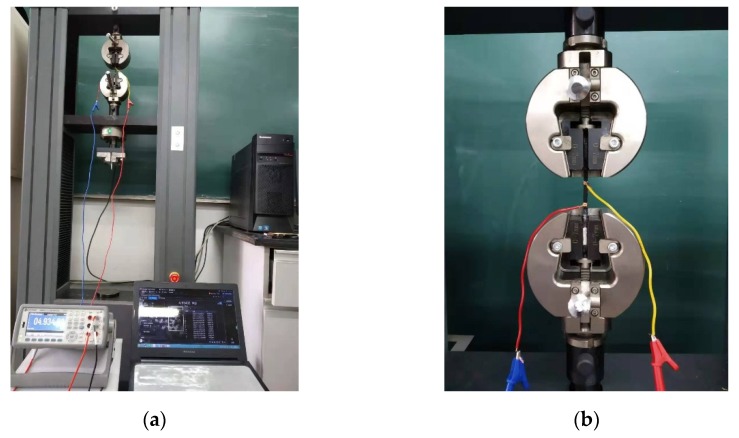
(**a**) Experimental setup for the piezoresistive sensing test and data measurement system, which included DMM and a data acquisition module connected to a computer, (**b**) Enlarged image showing composite sample mounted in UTM during the piezoresistive sensing test.

To examine the piezoresistive characteristics of the composites, silver paste was coated on the circumferences at two sides of the gauge length range of the specimen at intervals of 2 cm. The sample was mounted in a universal testing machine (UTM) (Jinan Fangyuan Testing Instrument Co., Ltd., China) as shown in Figure 2, and uniaxial tensile cyclic loads were applied by the UTM to the specimens at a displacement speed of 1 mm/min. The maximum tensile force was set to 0.32 kN (it was varied slightly in some samples due to differences in thickness and width), which produced stress of approximately 15 MPa. During the test, the change in electrical resistance, force, and displacement over time were collected in computers connected to the multimeter or the UTM. In addition, the change in electrical resistance was determined by the following formula [29].

(2) $$R_{f}=\frac{\Delta R}{R_{0}}=\frac{R_{i}-R_{0}}{R_{0}}$$

where $R_{f}$, $R_{i}$, and $R_{0}$ indicate electrical resistance change rate (fractional resistance change), instant resistance, and initial resistance, respectively [29].

## 3. Results

### 3.1. Elastic Modulus under Tensile Loading

**Figure 3:**
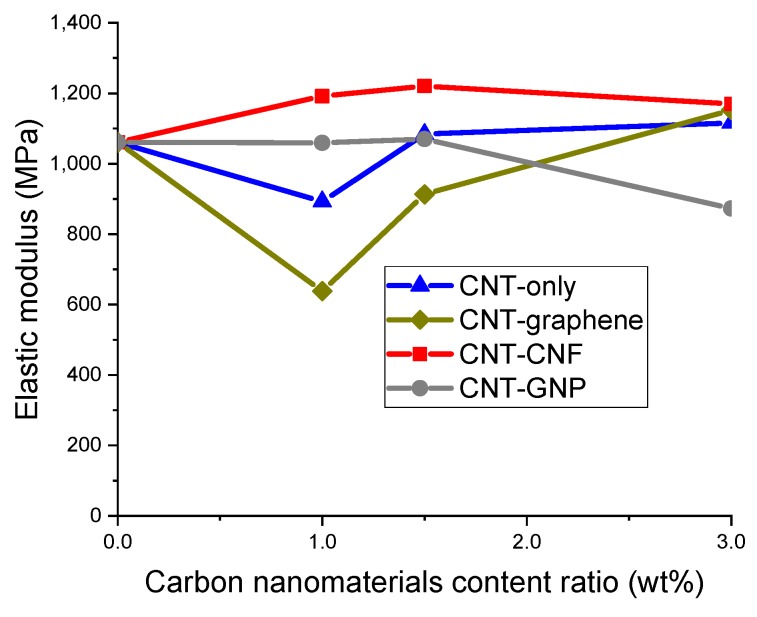
Elastic modulus of the epoxy-based composites incorporating CNT-only, CNT–graphene, CNT–CNF, or CNT–GNP, determined in tensile loading tests and plotted as a function of CNM content ratio.

Figure 3 shows the elastic modulus of CNM-embedded epoxy composites when tensile stress and strain were measured in a linear elastic region. In CNT–CNF composites and CNT-only composites, enhancements in elastic modulus were accomplished in most of the sample types with varied CNM content ratios, owing to the incorporation of CNT or CNT–CNF. In view of the results from the two sample groups, one-dimensional CNM appeared to be more advantageous than two-dimensional CNM for enhancing the elastic modulus. Aguilar-Bolados et al. (2017) and Li et al. (2013) demonstrated that CNT brought an enhancement to the elastic modulus that was greater than that gained by graphene oxide or GNP when the CNMs were embedded in polymer matrices with identical content ratios [1,26].

The enhancement of the elastic modulus was prominent in the composites incorporating CNM at 3%. The enhancement ratios were calculated as 10.2%, 8.6%, and 5.2% due to the incorporation of CNT–CNF, CNT–graphene, and CNT-only, respectively. The enhancement ratios obtained by CNT–CNF composites and CNT–graphene composites were superior to that from CNT-only composites, which demonstrated synergistic effects attributed to hybridized networks of CNT–CNF and CNT–graphene in terms of the elastic modulus.

In epoxy composites incorporating CNT–CNF, the hybrid effect was more pronounced than in composites incorporating other types of CNM in terms of the elastic modulus. The composite incorporating 1% CNT–CNF showed an elastic modulus 33.6% greater than that of the 1% CNT-only composite and the composites incorporating 1.5% CNT–CNF and 3% CNT–CNF exhibited enhancement ratios of 12.6% and 4.8%, respectively.

### 3.2. Electrical Conductivity

**Figure 4:**
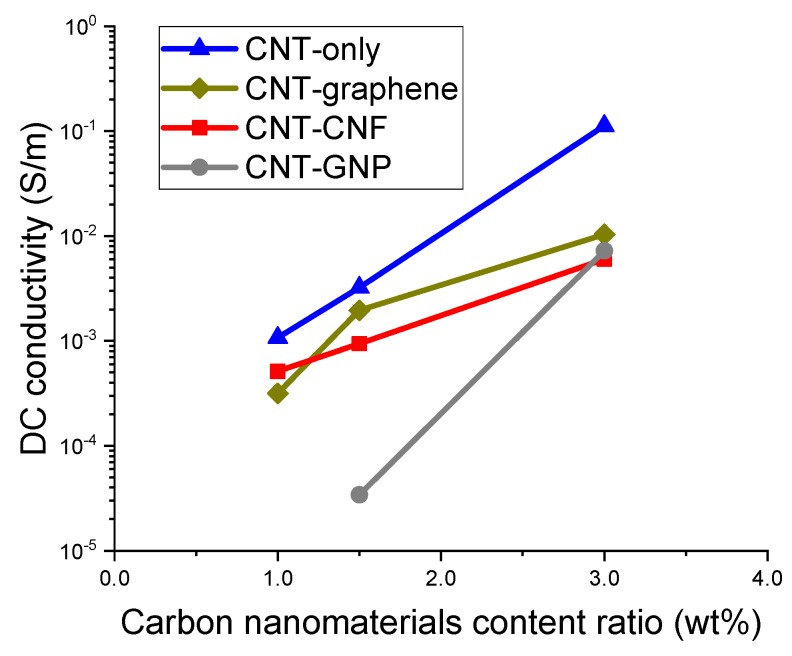
Electrical conductivity of the epoxy-based composites incorporating CNT-only, CNT–graphene, CNT–CNF, or CNT–GNP, determined by means of two-probe method and plotted as a function of CNM content ratio.

The electrical conductivity of the composites incorporating CNT only, CNT–graphene, CNT–CNF, and CNF–GNP was determined with DC conductivity, and the results are presented in Figure 4. The composites incorporating graphene only or GNP only with content ratios of 1% and 1.5% showed unmeasurable values in the multimeter, which means their electrical conductivity was less than 1 × 10^−6^ S/m. In addition, the composite incorporating CNT–GNP with a content ratio of 1% also did not show a measurable value. For the composites incorporating CNF only with content ratios of 1% and 1.5%, the conductivity was determined to be 4.2 × 10^−5^ and 3.3 × 10^−4^ S/m, respectively.

In Figure 4, the conductivity of the four composite types increased with an increase in the content ratio of the CNMs. The composites incorporating CNT only demonstrated the best electrical conductivity, followed by the CNT–graphene, CNT–CNF, and CNT–GNP composites. The superiority of the conductivity of the CNT-only composites and the CNT–graphene composites is attributed to the intrinsic electrical conductivity of CNT and graphene surpassing that of CNF and GNP [6,30].

Percolation threshold refers to a mean value of a range of content ratios where a remarkable increase of electrical conductivity occurs in the relationship between the conductivity and the content ratio [15,19]. The percolation phenomenon resulted from a formation of connected networks of conductive fillers, CNMs in the present study, in an entire polymer matrix. The range of percolation threshold of each composite type can be presumably determined on the basis of threshold values found in the literature.

Various experimental works have demonstrated that polymer composites incorporating CNT, GNP, or graphene oxide showed conductivity greater than 1 × 10^−5^ S/m provided that the content ratio of CNM exceeded the percolation threshold [12,26,31,32,33]. As a result, it can be said that conductive networks of all the composites shown in Figure 4 were formed beyond the percolation threshold. In addition, it is noteworthy that the percolation thresholds of the CNT-only, CNT–graphene, and CNT–CNF composites are located between the content ratio of 0% and 1%. For the composite type incorporating CNT–GNP, the percolation threshold is located between the content ratio of 0% and 1.5%, which is greater than the other types.

Based on the findings relevant to the range of percolation threshold, it can be stated that the epoxy-based nanocomposites incorporating over 1% CNM (1.5% for CNT–GNP) possess over-saturated CNM networks. While multiple existing publications demonstrated that over-saturated CNM networks led to a deteriorated gauge factor, some publications showed enhancement in time-based sensitivity, known as peak shift. In other words, if the CNM content ratio is close to the percolation threshold, a prominent gauge factor can be achieved but the time at the electrical resistance peak would not match the time at the applied stress peak. However, in the case of the composites incorporating over-saturated CNM networks, although a modest gauge factor can be produced, the time at the resistant peak would consistently match the time at the stress peak. A detailed discussion of the piezoresistive sensing characteristics of the fabricated composites will be dealt with in Section 3.4.

### 3.3. SEM Images

**Figure 5:**
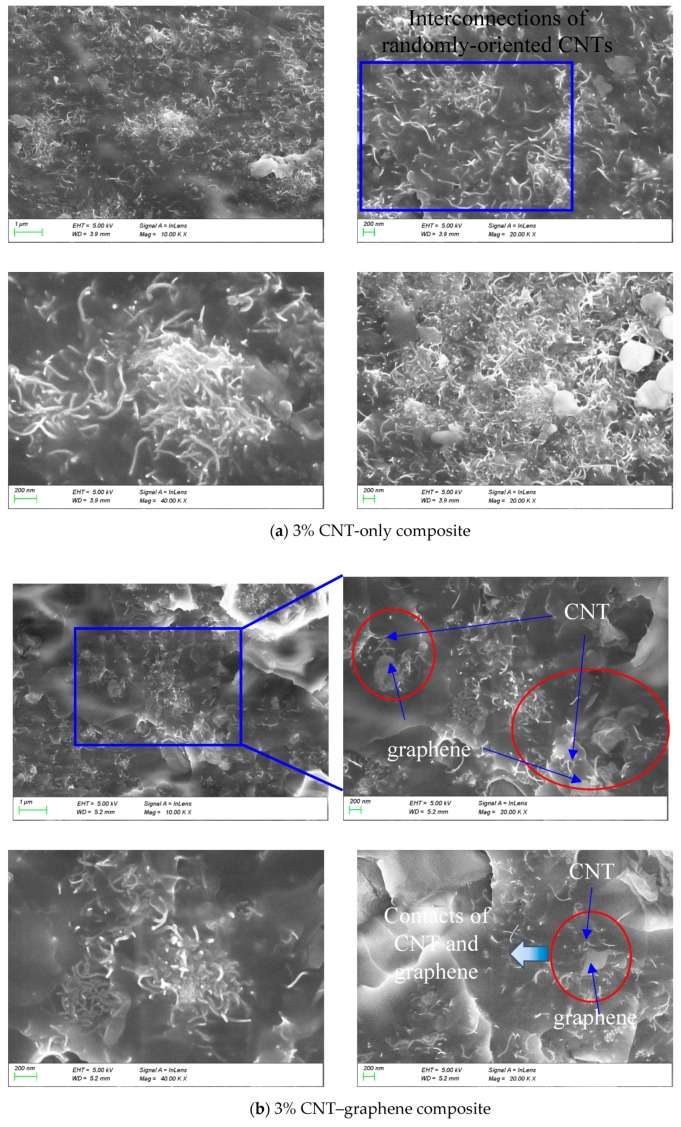

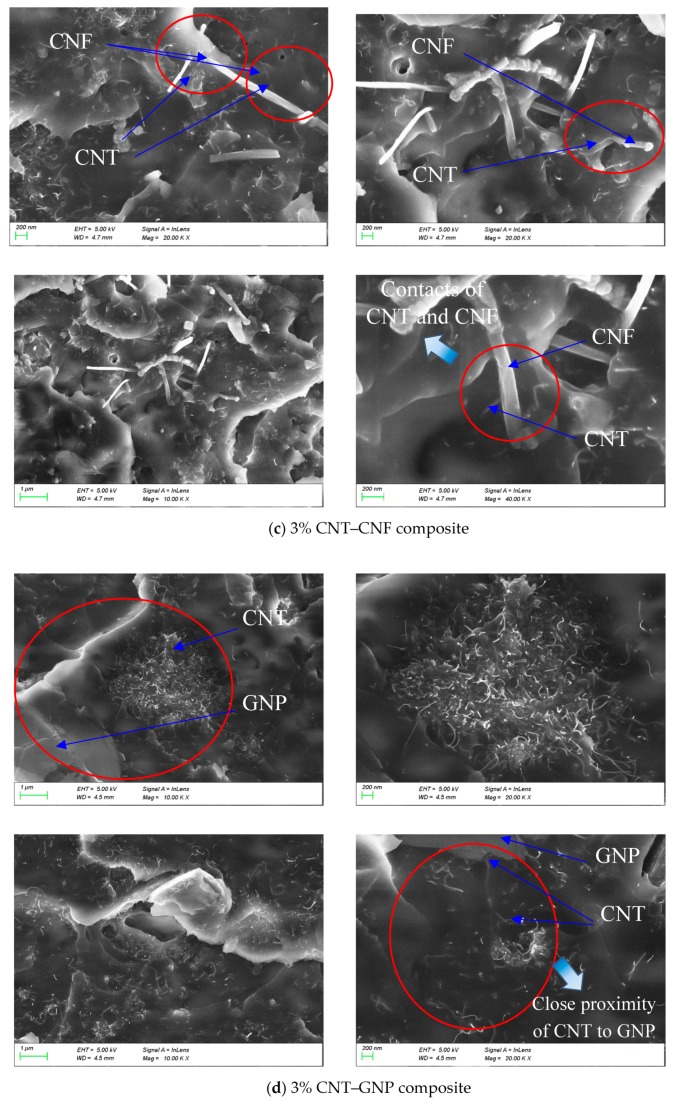
SEM images observed in varied magnifications and obtained from fractured surfaces of (**a**) the epoxy-based composites incorporating 3% CNT-only, (**b**) the epoxy-based composites incorporating 3% CNT–graphene, (**c**) the epoxy-based composites incorporating 3% CNT–CNF, and (**d**) the epoxy-based composites incorporating 3% CNT–GNP.

Figure 5a shows SEM images of 3% CNT-only composites. It can be seen in the figure that the CNT was distributed and embedded in the matrix. Figure 5b shows SEM images obtained from 3% CNT–graphene epoxy composites with CNT and graphene distributed in the matrix. The graphene is identified by the dimension of a nano-sheet provided by the manufacturer and also identified by referring to transmission electron microscopic (TEM) and atomic force microscopic (AFM) images of graphene shown in the literature [13,25]. It is noteworthy that the CNTs with a high aspect ratio wrap around the bendable graphene, and the interconnections were confirmed, which led to enhancements in mechanical/electrical properties.

Figure 5c shows SEM images of the composite incorporating 3% CNT–CNF, where it can be seen that CNT and CNF are randomly distributed in epoxy resin. According to the size provided by the manufacturer, CNFs are much larger than CNTs in length and diameter, which means they are distinguishable. CNFs were shown with a skeleton-shape in the epoxy resin, and CNTs were filled between CNFs, which displayed hierarchical network structures. In some local parts, CNTs and CNFs were interconnected with one another. These hierarchical network structures and interconnections of CNFs and CNTs are beneficial to improving the mechanical/electrical characteristics of the CNT–CNF composites.

In the procedure of CNT and CNF dispersion implemented by means of a three-roll milling machine, entangled CNTs and CNFs were separated due to the shear forces of the milling process. The separation of CNTs and CNFs yielded space between the two materials, and allowed inflow of epoxy resin. The thickness of the epoxy resin filled between the CNTs and CNFs was different due to the different dispersion degree and random position of CNTs and CNFs, which can be observed in the SEM images. With lapse of time, the epoxy resin between the CNTs and CNFs solidified, which led to binding between the CNTs and CNFs.

Figure 5d displays an SEM image of the composite incorporating 3% CNT–GNP. Individual CNTs and CNT clusters were found, and a planar GNP with a length greater than 4 μm was identified. While the two materials were adjacent to one another, contacts were not distinct.

### 3.4. Piezoresistive Characteristics of the Composites Subjected to Repetitive Tensile Loadings

**Figure 6:**
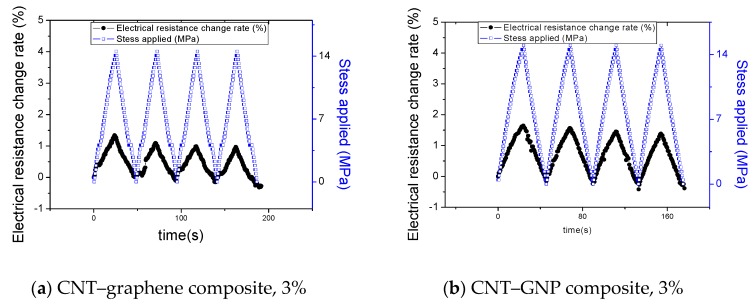
Electrical resistance change rates and applied stress change curve of composites determined with the lapse of time under repetitive tensile loadings: (**a**) the epoxy-based composites incorporating 3% CNT–graphene and (**b**) the epoxy-based composites incorporating 3% CNT–GNP (Piezoresistive responses of other composite types can be found in Figure S4).

Figure 6 and Figure S4 present the piezoresistive characteristics of the composites under cyclic tensile loading. In most of the sample types, a linear increase or decrease in the electrical resistance change rate was exhibited in response to loading or unloading of tensile force, which can be found in typical thermosetting polymer composites incorporating carbon materials [14].

**Figure 7:**
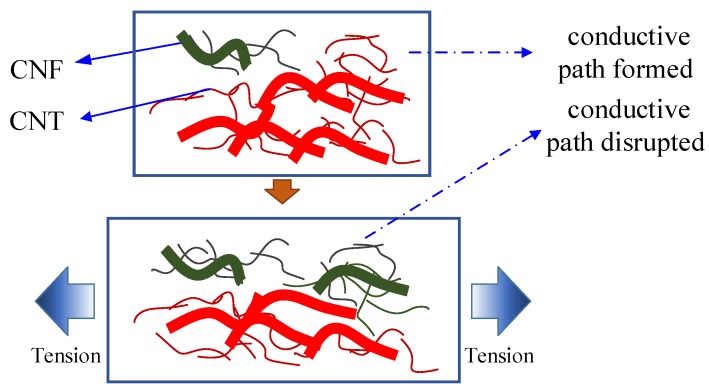
The piezoresistive sensing mechanism in the epoxy-base composites incorporating CNT–CNF under tensile loading.

The epoxy-based composites incorporating CNMs have a positive Poisson’s ratio. When the tensile load is applied, the composites elongate longitudinally and shorten transversely. With an increase of applied load, the distance between the CNMs of the samples was changed. For the longitudinal direction, the distance between CNMs with a large portion of CNMs increased, which caused disruption of conductive paths of the CNMs. For the transverse direction, the distance between CNMs with a relatively small portion of CNMs compared to CNMs portion in the longitudinal direction decreased, which might produce contacts and conductive paths of CNMs. In view of the global conductive network of CNMs, the aforementioned phenomena result in an increase of electrical resistance of the composites since a relatively large portion of conductive paths of CNMs was disrupted (Figure 7). When the tensile force is unloaded, an opposite phenomenon in the conductive paths takes place.

Aside from the piezoresistive characteristics of the composites, it is worth discussing the variations in initial electrical resistance at points where each loading cycle was resumed. The resistance at a resuming point of a loading cycle was lower than the first electrical resistance value (baseline) in all composite types. The tendency of decreasing from the initial resistance was also presented and discussed in multiple studies in the literature [13,32], and it can be explained as follows. Once a tensile loading cycle is completed, permanent deformation can be produced in the gauge length part of the composite. The deformation produces elongation in the longitudinal direction and shrink in the transverse direction of the gauge length part because the composite has a positive Poisson’s ratio [34]. Due to the shrinkage, compressive stress is produced in the transverse direction, and this leads to reorientation/reconfiguration of the three-dimensional CNM networks toward denser structures in the network [34].

**Figure 8:**
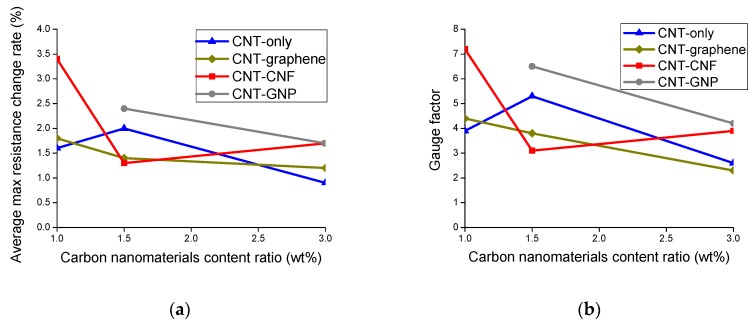
(**a**) Maximum resistance change rate, and (**b**) gauge factor of the epoxy-based composites incorporating CNT-only, CNT–graphene, CNT–CNF, or CNT–GNP, determined as a function of CNM content ratio and derived from the piezoresistive sensing test.

Variations in the average maximum resistance change rate and gauge factor are presented as a function of content ratio of the CNMs in Figure 8a,b, respectively. The average maximum resistance change rate denotes averaged peak values of the electrical resistance change rate calculated from three replicated samples of each sample type. Gauge factor refers to a ratio of the maximum resistance change rate to strain at a maximum tensile load ($R_{f}/\varepsilon$), and this was derived by calculating average values from three replicated samples of each sample type [29,32]. Both the average maximum resistance change rate and gauge factor were relatively large as the content ratio of CNMs became adjacent to the percolation threshold [13,35]. For example, the CNT–CNF content ratio of 1% or CNT–GNP content ratio of 1.5%, which are close to the percolation threshold, resulted in relatively large values in terms of the two factors. On the other hand, the two factors tended to decrease as the content ratio moved further from the percolation threshold; for example, it was shown at a content ratio of 3%. These can be explained with the factors underlying the piezoresistive phenomenon: (1) disruption and formation of CNMs, and (2) change in distance between CNMs [14]. In the case where the tensile load was applied to a composite with a CNM content ratio close to the percolation threshold (e.g., CNT–GNP content ratio of 1.5%), the proportion of disrupted conductive path and loss of tunneling effect due to enlargement of the distance between CNMs was significant for the entire conductive path in the network (Figure S5a). In contrast, in the case where the tensile load was applied to a composite with a CNM content ratio far greater than the percolation threshold (e.g., CNM content ratio of 3%), due to dense conductive paths in the network, the proportion of the disrupted conductive paths and the loss of tunneling effect were not significant for the entire conductive path (Figure S5b).

Accordingly, as shown in Figure 8, both the average maximum resistance change rate and the gauge factor displayed decreasing tendencies as the CNM content ratio increased further from the percolation threshold. This tendency stands in line with the results of multiple studies found in the literature [7,15,20].

In addition, for the composites in which the CNM network was sufficiently formed with a content ratio of 3%, the greatest gauge factor was accomplished by the CNT–GNP composite, followed by the CNT–CNF composite, and this could not be achieved by the CNT-only composite.

The hybrid effect was the most pronounced in the epoxy composite incorporating CNT–GNP in terms of the gauge factor. The composite with 1.5% CNT–GNP showed a gauge factor 21.1% greater than that of the 1.5% CNT-only composite and the composites incorporating 3% CNT–GNP exhibited an enhancement ratio of 64.7%. The superior gauge factor of the hybridized-network of CNT–GNP can be explained with geometrical features of the CNTs and GNP.

To describe the piezoresistive characteristics of the composites in relation to the geometrical features of the CNMs, excluded volume theory was employed [19]. Excluded volume refers to an unoccupied space that is located adjacent to the CNMs and which does not contain them [19]. When packing rod-shaped or planar materials into limited three-dimensional space, materials with particular shapes cannot be packed densely, only loosely, due to the geometrical features of the CNMs, which represents a large amount of the excluded volume [19]. The excluded volume is calculated as follows, taking into account the material shape and dimensions [19,36].

(3) $$Excluded\;volume=\{\begin{array}{cc} \left(\frac{\pi }{2}\right)L^{2}d+2\pi d^{2}L+\left(\frac{4}{3}\right)\pi d^{3} & \left(\mathrm{rods}\right)\\ \pi^{2}r^{3} & \left(\mathrm{disks}\right) \end{array}$$

where *L* and *d* denote the length and diameter of the rod type CNMs, and *r* denotes the radius of the disk type CNMs [19,36].

The CNTs and CNFs used in this study were considered rod types, but the graphene and GNP were classified as disk types. In addition, the values L and d of each CNM were used for mean values of the material dimensions provided by the manufacturer. The values of the excluded volume and the dimensions of the CNMs used in the calculations are listed in Table 3. As shown, the excluded volume of GNP was the largest, followed by that of CNF, graphene, and CNT. The large excluded volume signifies that the CNMs were loosely packed, thereby accounting for relatively less contact and smaller tunneling effect of CNMs [19]. For these reasons, the disruption of conductive paths and loss of tunneling effect within the network under tensile loading resulted in a significant impact on the conductivity of the entire network [19]. In the present study, the gauge factor of the CNT–GNP composite was the greatest, followed by that of the CNT–CNF composite at a CNM content ratio of 3%, where the CNM network was formed with a sufficient amount beyond the percolation threshold. It is noteworthy that the gauge factor results at a content ratio of 3% was closely related to the calculated excluded volume [19].

**Figure 9:**
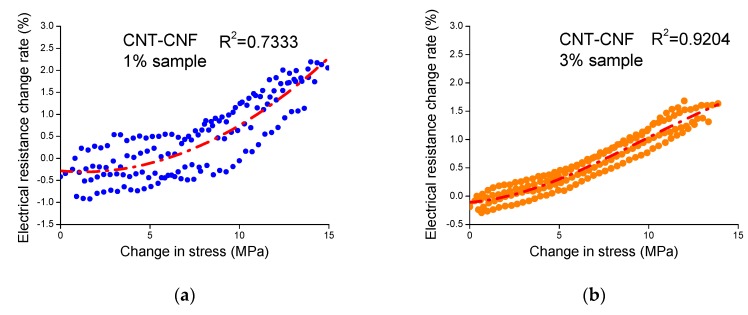
Scattered points derived from the relationship between the electrical resistance change rate and stress change of (**a**) the epoxy-based composites incorporating 1% CNT–CNF and (**b**) the epoxy-based composites incorporating 3% CNT–CNF during the piezoresistive sensing test.

**Figure 10:**
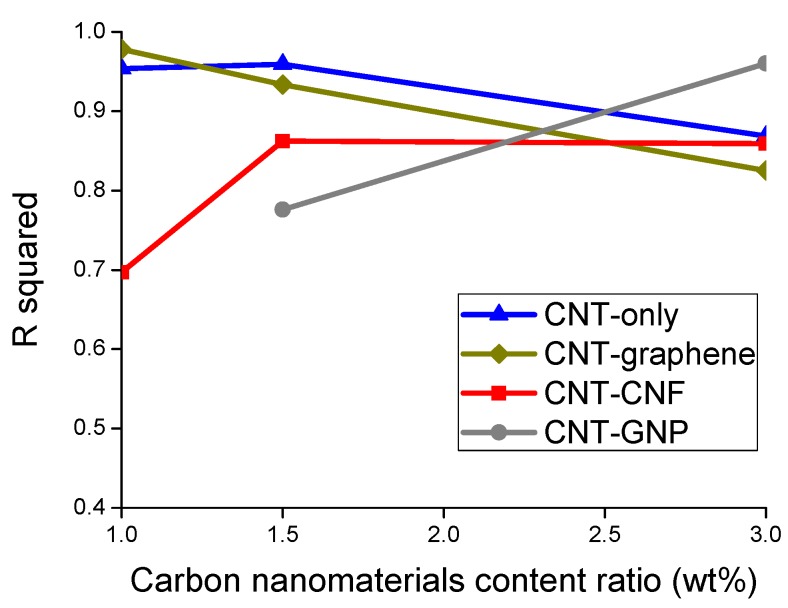
The coefficient of determination, R^2^ (R squared), of the regression derived from the relationship between the electrical resistance change rate and stress change during the piezoresistive sensing test of the epoxy-based composites incorporating CNT-only, CNT–graphene, CNT–CNF, or CNT–GNP.

To assess the sensing stability of the composites, a cubic polynomial regression fit to a data set of stress versus electrical resistance change rate was derived, and the coefficient of determination, R^2^ (R squared) of the regression was calculated. The R^2^ implies the extent of data scattering graphically represented in the relationship between stress and electrical resistance change rate. For example, data points determined from the relationship between stress and electrical resistance change rate were scattered to a relatively large degree, as shown in Figure 9a, and this case yielded relatively little R^2^. On the other hand, data points in Figure 9b were scattered to a relatively small degree, thereby producing a relatively large R^2^. Figure 10 presents R^2^ values averaged from the values of three replicated samples of each sample type. Most of the sample types, except for the CNT–CNF 1% composite and the CNT–GNP 1.5% composite, showed R^2^ values greater than 0.83, which signifies stable and reliable sensing characteristics. Referring to both R^2^ and gauge factor, the 3% CNT–GNP composite and 1.5% CNT-only composite exhibited relatively high gauge factors as well as relatively high R^2^ values, which can lead to feasible application of piezoresistive sensing.

**Figure 11:**
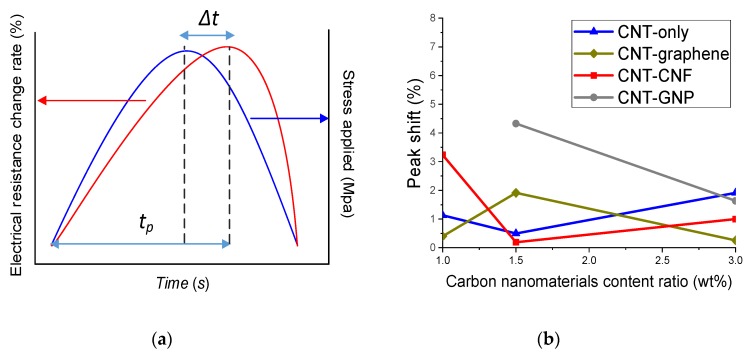
(**a**) Illustrative descriptions of peak shift, which is expressed as a percentage obtained by dividing the time interval of the resistant peak and the applied stress peak (Δ*t*) by the time difference (*t_p_*) between the initiating point and the peak point of the electrical resistance change rate curve, (**b**) peak shift values derived from the epoxy-based composites incorporating CNT-only, CNT–graphene, CNT–CNF, or CNT–GNP.

To assess and compare the time-based sensitivity, the peak shift of the fabricated nanocomposites was determined [37]. The peak shift is expressed as a percentage obtained by dividing the time interval of the resistant peak and the applied stress peak (Δ*t*) by the time difference (*t_p_*) between the initiating point and the peak point of the electrical resistance change rate curve [37]. An illustrative description of Δ*t* and *t_p_* is given in Figure 11a, and an equation of the peak shift is given in Equation (4) [37].

(4) $$Peak\;shift\;\left(\%\right)=\frac{\Delta t}{t_{p}}\times 100$$

The low value of peak shift represents that the resistance curve promptly changes in accordance with change of the applied stress. Figure 11b presents the peak shift values derived from the CNM-incorporated epoxy composites. Each value was calculated by averaging the peak shift of three replicated samples of each type of composite. Recalling the sensing stability (R squared) results (Figure 10) and maximum electrical resistance rate, it was found that composite types exhibiting relatively low sensing stability or relatively high maximum electrical resistance rate brought a large peak shift, which means poor time-based sensitivity. This correlation was conspicuous in the CNT–CNF composite and the CNT–GNP composites. In addition, it is noteworthy that in the epoxy-based nanocomposites incorporating 3% CNM, which possess a sufficient amount of CNM beyond the percolation threshold, the composites incorporating the hybridized networks of CNM showed superior sensing sensitivity with less peak shift than the composites incorporating only CNTs.

## 4. Concluding Remarks

In the present study, polymer composites fabricated with the hybridized CNMs network were examined in terms of mechanical, electrical and piezoresistive characteristics. Specifically, the polymer composites were fabricated with hybridized CNM networks, such as CNT–graphene, CNT–CNF, or CNT–GNP, in an effort to overcome limited mechanical, electrical and piezoresistive characteristics that composites incorporating only a single type of CNM possess. The experimental results of the present study can be summarized as follows:

1. In the cases of composites with CNMs of 3%, elastic modulus of the CNT–CNF composite and CNT–graphene composite were greater than that of the CNT-only composite, which indicated synergistic effects attributed to the hybridized CNMs network.
2. In the results of electrical conductivity of the composites, the percolation threshold of the CNT-only, CNT–graphene, and CNT–CNF composites ranged from 0% to 1%, but the CNT–GNP composite had a percolation threshold at a content ratio between 0% and 1.5%, which is larger than that of the other types of composites.
3. SEM images showed that CNMs were distributed in the epoxy matrix and demonstrated interconnections of CNT–graphene and CNT–CNF, which led to enhancements in mechanical/electrical characteristics.
4. It was found that gauge factor tended to decrease as the CNM content increased further beyond the percolation threshold. For the composites where the CNM networks were sufficiently formed with a content ratio of 3%, the greatest gauge factor was accomplished by the CNT–GNP composite, followed by the CNT–CNF composite, and this could not be achieved by the CNT-only composite. The superiority of the two types of composites in gauge factor was explained with the excluded volume theory.
5. The 3% CNT–GNP composite and 1.5% CNT-only composite exhibited relatively high R^2^ values, which was related to sensing stability, as well as a relatively high gauge factor.

## Figures and Tables

**Table 1:** Physical properties and dimensions of the carbon nanomaterials.

CNM	Diameter or Thickness	Layers	Purity	Specific Surface Area (m^2^/g)	True Density (g/cm^3^)	Length or Width (μm)	Electrical Conductivity (S/cm)
CNT	<8 nm (outer diameter) 2–5 nm (inner diameter)		>98%	>350	~2.1	10–30	>100
Graphene	0.55–1.2 nm (thickness)	1–5	>99%	>500		0.5–3	184.8
CNF	0.15–0.2 μm		99.9%	300	~2.0	10–30	
GNP		<30	>90%			2–16	6.67

**Table 2:** Distance of Gaps I and II adjusted in each pouring and withdrawal cycle.

Order	Gap I Distance (μm)	Gap II Distance (μm)
1	60	40
2	40	20
3	20	15
4	15	10
5	10	5
6	5	5
7	10	5
8	5	5
9	10	5
10	5	5
11	3	2
12	2	1

**Table 3:** Excluded volume calculated with dimensions of carbon nanomaterials.

	*d* (μm)	*L* (μm)	Excluded Volume (μm^3^)
CNT	0.008	20	5.0
Graphene	1.75		6.6
CNF	0.17	20	110.4
GNP	9		898.5

## Supplementary Materials

The following are available online at https://www.mdpi.com/1424-8220/20/7/2094/s1, Figure S1: A SEM image of as-received CNTs provided by the manufacturer., Figure S2: A SEM image of as-received graphene provided by the manufacturer., Figure S3: SEM images of as-received graphite nanoplatelet provided by the manufacturer., Figure S4: Electrical resistance change rates and applied stress simultaneously determined under repetitive tensile loadings., Figure S5: Schematics of carbon nanomaterials network at (**a**) a content ratio adjacent to percolation threshold and (**b**) a content ratio far greater than percolation threshold.
