# Supplementary material for: Mechanical, Electrical, and Piezoresistive Sensing Characteristics of Epoxy-Based Composites Incorporating Hybridized Networks of Carbon Nanotubes, Graphene, Carbon Nanofibers, or Graphite Nanoplatelets
Source: Sensors (Basel). 2020 Apr 8;20(7):2094. doi: 10.3390/s20072094 (PMC7180708; doi:10.3390/s20072094)
Supplement: Supplementary file 1 [file sensors-20-02094-s001.pdf]

sensors

MDPI

Article

Mechanical, Electrical, and Piezoresistive Sensing Characteristics of Epoxy-Based Composites Incorporating Hybridized Networks of Carbon Nanotubes, Graphene, Carbon Nanofibers, or Graphite Nanoplatelets

Supplementary Figures

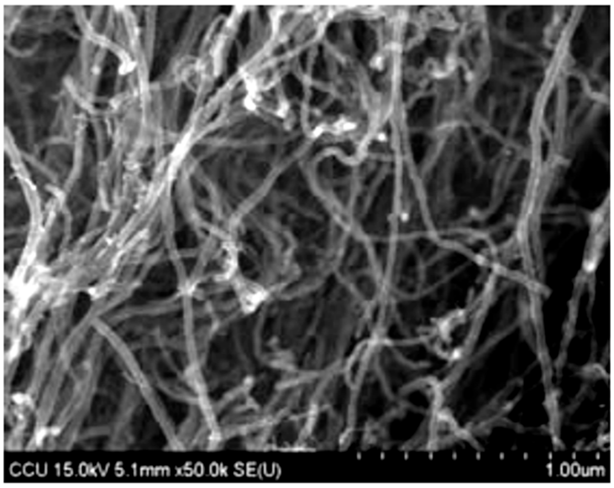

Figure S1. A SEM image of as-received CNTs provided by the manufacturer.

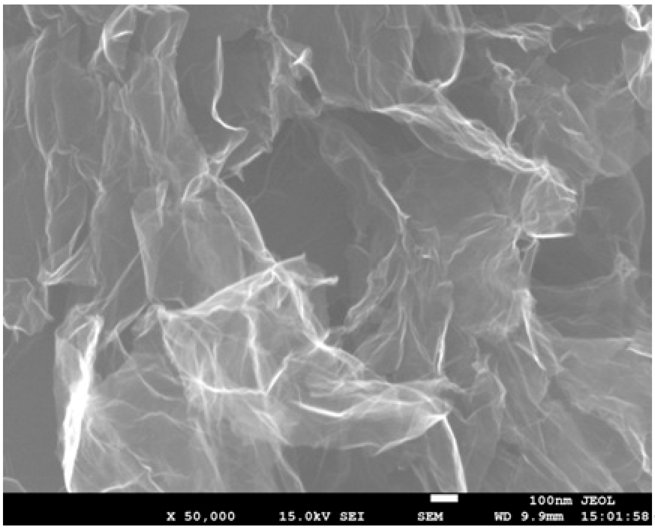

Figure S2. A SEM image of as-received graphene provided by the manufacturer.

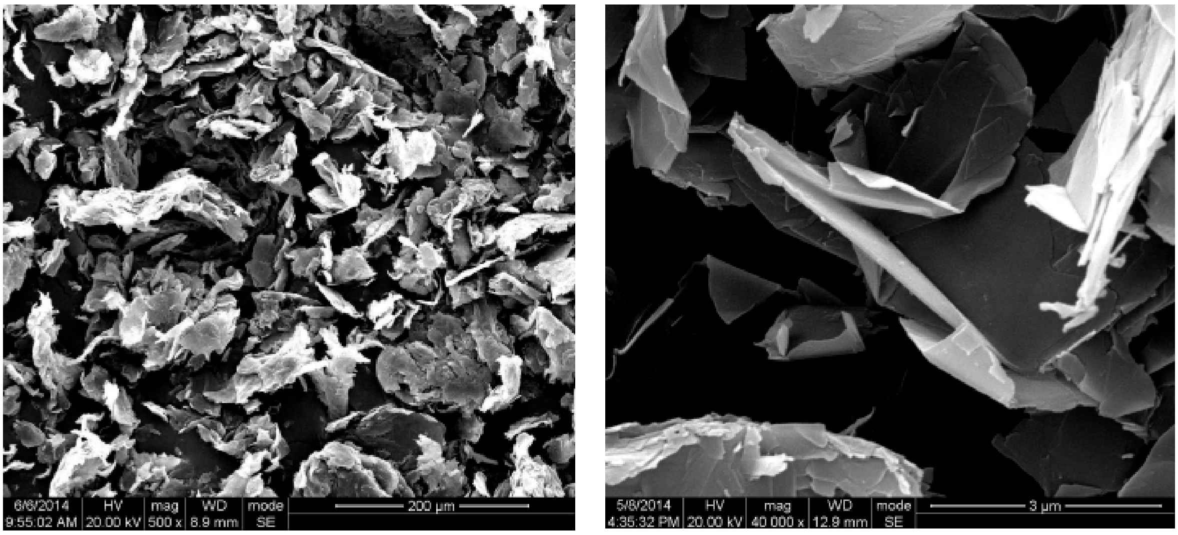

(**a**) (**b**)

Figure S3. SEM images of as-received graphite nanoplatelet provided by the manufacturer.

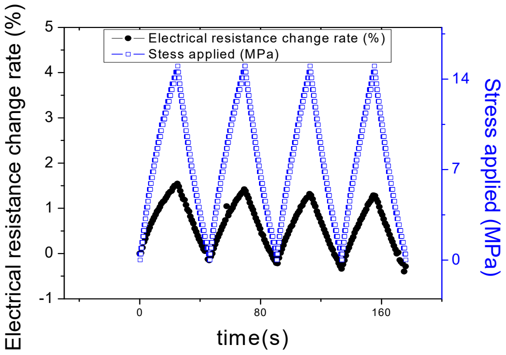

(**a**) CNT-only composite, 1%

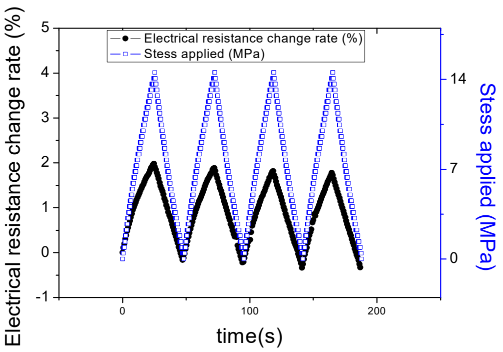

(**b**) CNT-only composite, 1.5%

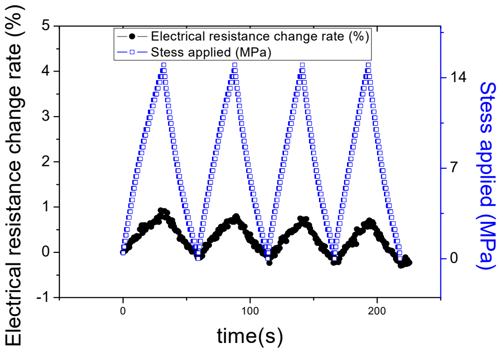

(**c**) CNT-only composite, 3%

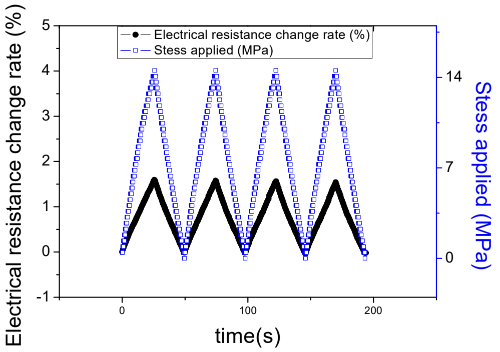

(**d**) CNT–graphene composite, 1%

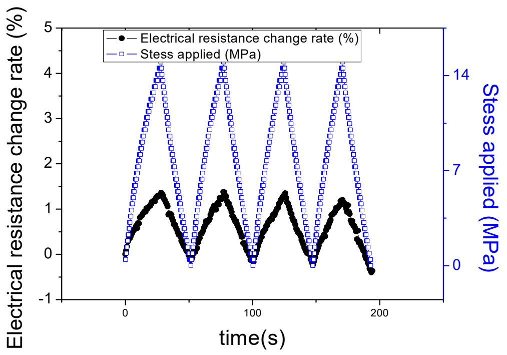

(**e**) CNT–graphene composite, 1.5%

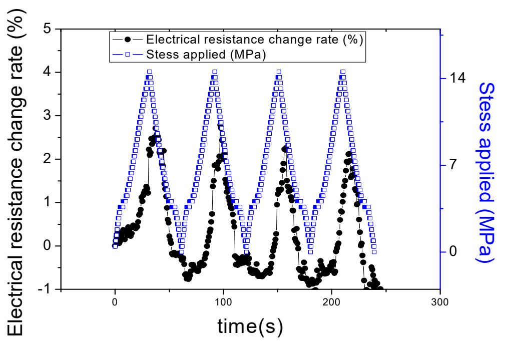

(**f**) CNT–CNF composite, 1%

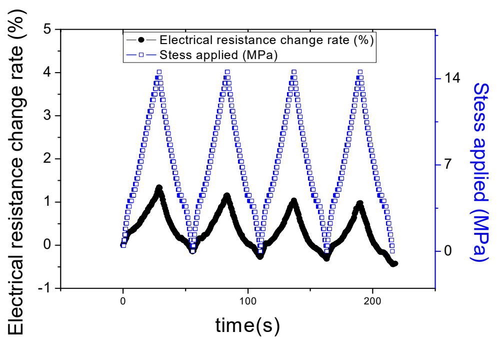

(**g**) CNT–CNF composite, 1.5%

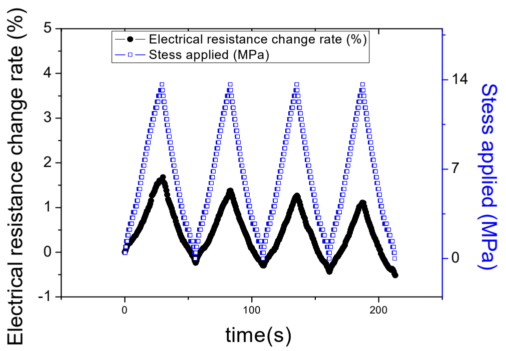

(**h**) CNT–CNF composite, 3%

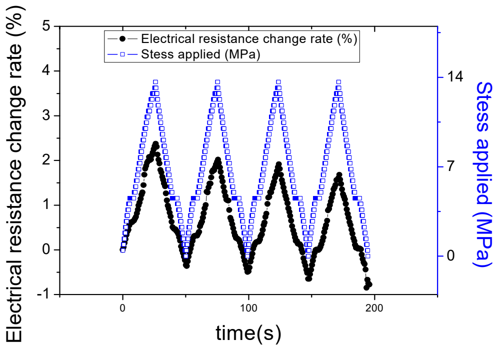

(**i**) CNT–GNP composite, 1.5%

Figure S4. Electrical resistance change rates and applied stress simultaneously determined under repetitive tensile loadings.

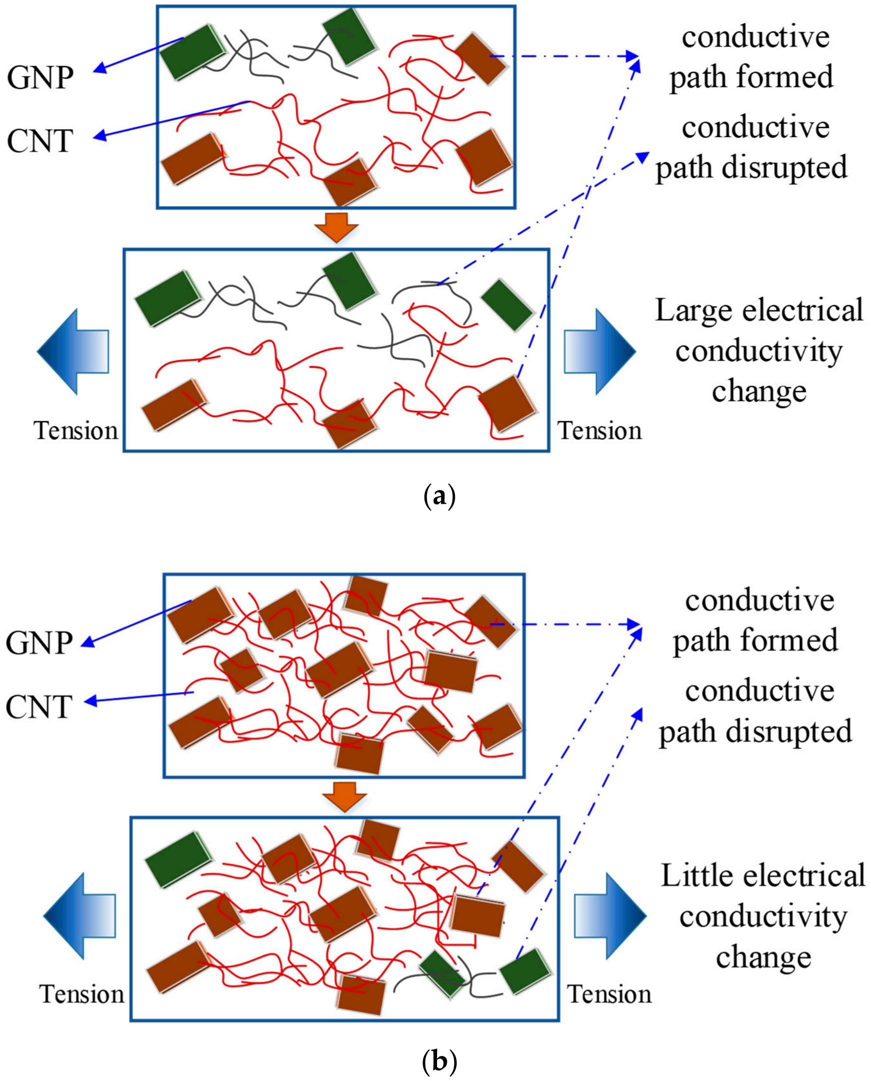

Figure S5. Schematics of carbon nanomaterials network at (**a**) a content ratio adjacent to percolation threshold and (**b**) a content ratio far greater than percolation threshold.

© 2020 by the authors. Licensee MDPI, Basel, Switzerland. This article is an open access article distributed under the terms and conditions of the Creative Commons Attribution (CC BY) license (http://creativecommons.org/licenses/by/4.0/).
